# Influence of Tuning Fork Resonance Properties on Quartz-Enhanced Photoacoustic Spectroscopy Performance

**DOI:** 10.3390/s19183825

**Published:** 2019-09-04

**Authors:** Huadan Zheng, Haoyang Lin, Lei Dong, Yihua Liu, Pietro Patimisco, John Zweck, Ali Mozumder, Angelo Sampaolo, Vincenzo Spagnolo, Bincheng Huang, Jieyuan Tang, Linpeng Dong, Wenguo Zhu, Jianhui Yu, Zhe Chen, Frank K. Tittel

**Affiliations:** 1 Key Laboratory of Optoelectronic Information and Sensing Technologies of Guangdong Higher Education Institutes, Department of Optoelectronic Engineering, Jinan University, Guangzhou 510632, China; 2 State Key Laboratory of Quantum Optics and Quantum Optics Devices, Institute of Laser Spectroscopy & Collaborative Innovation Center of Extreme Optics, Shanxi University, Taiyuan 030006, China; 3 Department of Electrical and Computer Engineering, Rice University, Houston, TX 77005, USA; 4 PolySense Lab-Dipartimento Interateneo di Fisica, Politecnico di Bari and Università degli Studi di Bari, Via Amendola 173, 70126 Bari, Italy; 5 Department of Mathematical Sciences, The University of Texas at Dallas, Richardson, TX 75080, USA

**Keywords:** quartz tuning fork, custom tuning fork, photoacoustic spectroscopy, quartz-enhanced photoacoustic spectroscopy

## Abstract

A detailed investigation of the influence of quartz tuning forks (QTFs) resonance properties on the performance of quartz-enhanced photoacoustic spectroscopy (QEPAS) exploiting QTFs as acousto-electric transducers is reported. The performance of two commercial QTFs with the same resonance frequency (32.7 KHz) but different geometries and two custom QTFs with lower resonance frequencies (2.9 KHz and 7.2 KHz) were compared and discussed. The results demonstrated that the fundamental resonance frequency as well as the quality factor and the electrical resistance were strongly inter-dependent on the QTF prongs geometry. Even if the resonance frequency was reduced, the quality factor must be kept as high as possible and the electrical resistance as low as possible in order to guarantee high QEPAS performance.

## 1. Introduction 

Photoacoustic spectroscopy (PAS) is a powerful technique for trace gas analysis and has been widely used in various fields of physics, chemistry, and biology [1,2,3]. In PAS, the photoacoustic signals are generated from intensity-modulated light absorption of target analytes, resulting in local heating and the generation of acoustic pressure waves, via no-radiative vibrational-translational (V-T) relaxation processes occurring in excited molecules. Highly sensitive microphones are used to transduce the weak acoustic waves intensity into electronic signals proportional to the gas target concentrations. A well-established variant of PAS is quartz enhanced photoacoustic spectroscopy (QEPAS), which was firstly demonstrated by Kosterev et al. [4,5] in 2002. Instead of a microphone, a quartz tuning fork (QTF) is employed as a compact transducer to detect the sound waves that are generated when a light beam is focused between the prongs of a QTF. Several types of laser sources such as light emitting diodes (LEDs), telecommunications diode lasers (TDLs), quantum cascade lasers (QCLs), inter-band cascade lasers (ICLs), optical parametric oscillators (OPOs), and terahertz (THz) QCL sources have been implemented in QEPAS sensors for trace gas analysis [6,7,8,9,10]. In most of the cases, standard QTFs, i.e., the ones mass-produced as timing elements in clocks and smartphones, have been employed. The standard QTFs are designed to have an extremely high-quality factor (Q factor), ~100,000 in vacuum and ~10,000 in air [6,7]. The QTF’s narrow response bandwidth Δ*f* of few Hz makes the QEPAS immune to the environmental acoustic noise, thus resulting in a high detection sensitivity. However, commercial QTFs are not ideal transducers for the detection of molecules with slow V-T relaxation rates. The comparison of QEPAS and conventional PAS was reported in our previous publication [11]. For the detection of molecules with fast V-T relaxation rates such as C_2_H_2_, the QEPAS shows better normalized noise equivalent absorption coefficient (NNEA) than conventional PAS. But for the detection of pure CO_2_, the performance of conventional PAS is betters. This is because the commercially available QTFs are designed and optimized for timing application with the resonance frequency of 2^15^ (32768) Hz and the slow de-excitation processes of some molecules cannot efficiently follow such a fast laser modulation frequency [12,13]. In addition, when light sources with a poor spatial beam quality (such as LEDs or THz QCLs) are employed in a QEPAS sensors, the 200–300 µm prong spacing of commercial QTFs represents a serious challenge for beam focalization between prongs. Indeed, the light blocked by prongs can result in undesirable background noise which can limit the ultimate detection sensitivity of the sensor [14]. To overcome these limitations, custom QTFs with low-resonance frequencies of a few kHz and prong spacings >700 µm have been proposed for QEPAS [8,14,15,16,17]. The photoacoustic signal *S* of QEPAS can be expressed as:

(1) $$S=KP_{L}Q\left(P\right)\alpha \left(P\right)\varepsilon \left(P,f_{0}\right)$$

where *P*_L_, *Q*, and *α*, are the laser power, the *Q* factor of the QTF resonance and the gas absorption coefficient, respectively. *ε(P, f*_0_*)* is the conversion efficiency of the absorbed optical power in sound and it is dependent on the gas pressure and the QTF resonance frequency *f*_0_ [13]. The sensor constant *K* represents the transduction efficiency of the QTF, i.e., the conversion of the acoustic pressure wave hitting the internal side of two prongs into transversal in-plane deflections. Hence, *K* depends on the position of the laser focalization point. Theoretically, the focused laser spot should be located where the maximum vibration amplitude occurs, i.e., between the two prongs and in correspondence of the antinode point of the vibration profile [12,18,19,20].

In this work, two standard QTFs with the same resonance frequency (32.7 KHz) but different geometries and two custom QTFs with lower resonance frequencies (2.9 KHz and 7.2 KHz) have been implemented in a QEPAS sensor system operating in the near-IR spectral range to detect water vapor in the lab air. A detailed analysis of the dependence of the QEPAS sensor performance on the QTF resonance properties have been discussed.

## 2. Quartz Tuning Fork Characterization 

**Figure 1:**
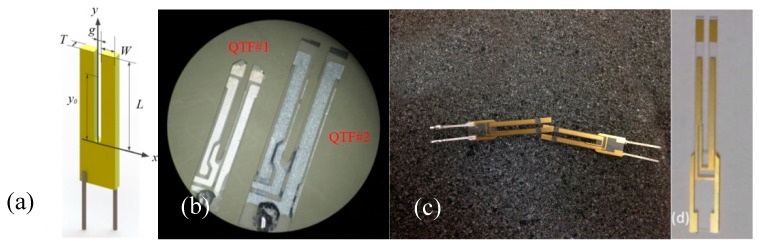
(**a**) Sketch and coordinate system of a quartz tuning fork (QTF). The origin of the *y*-axis is at the junction of the QTF; (**b**) picture of the commercial QTF#1 and #2; (**c**) picture of two samples of the custom QTF#3. (**d**) and a picture of the custom QTF#4.

The realization of QTFs was originally developed in the 1970s. Usually, commercial QTFs are etched using microelectronic clean room techniques starting from hundreds of micrometer thick Z-cut quartz wafers [21], with QTF prongs oriented along the *y*-axis, see Figure 1a. The electrodes, made of silver or gold, are deposited on adjacent sides of the prongs of the QTF. When one of the in-plane flexural modes is excited, the two prongs vibrate in the QTF planes, namely, the *xy*-plane. A picture of two commercial QTFs, named QTF#1 and QTF#2, employed in this work is shown in Figure 1b. For custom QTFs, a Z-cut quartz wafer with a 2˚ rotation along the *x*-axis was selected and standard photolithographic techniques were used to etch the QTFs. Chromium and gold patterns were photolithographically defined on both sides of the wafer. A three-dimensional structure was generated by chemical etching in a hydrogen fluoride solution, and finally the side electrodes were deposited by means of shadow masks [21]. A picture of two custom QTFs, named QTF#3 and QTF#4, is shown in Figure 1c,d. 

The geometric parameters of the four QTFs are listed in Table 1.

**Figure 2:**
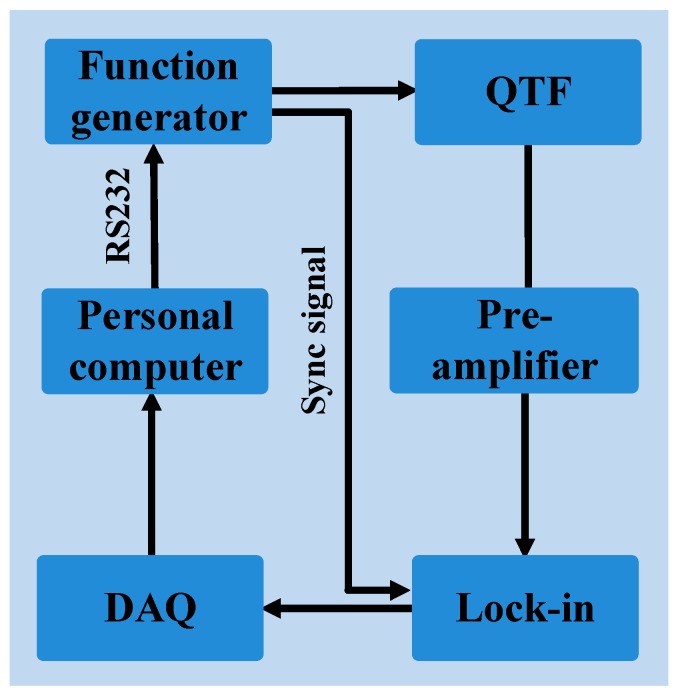
Circuit diagram employed for QTF characterization. QTF: quartz tuning fork, Lock-in: lock-in amplifier, DAQ: data acquisition.

The resonance frequency of the fundamental flexural mode of the QTF can be theoretically predicted by using the Euler–Bernoulli beam theory [7,8]. Assuming that each prong of the QTF behaves as a clamped beam, the frequencies of the flexural modes of a single beam are obtained by including a free-motion condition on one boundary of the beam and a clamped condition on the other end. Then the equation is solved for the propagation of a shear sound wave. Imposing these conditions, the fundamental resonance frequency of the QTFs is expressed by:

(2) $$f_{th}=\frac{\pi W}{8\sqrt{12}L^{2}}\sqrt{\frac{E}{\rho }}1.194^{2}$$

where *E* and *ρ* are the Young modulus and the density of quartz, respectively. The resonance frequency, as well as the quality factor and the electrical resistance of the QTFs, can be experimentally measured by electrical excitation, using the setup depicted in Figure 2. 

A function generator (Tektronix AFG3102) was used to provide a sinusoidal voltage excitation to the QTF. The generated piezocurrent passed through a current-to-voltage converter using an operational transimpedance amplifier. A lock-in amplifier (Standford SR830 DSP) was used to demodulate the QTF output signal at the excitation frequency. The frequency of the sine signal was scanned a step of 0.2 Hz to retrieve the resonance frequency profile of the QTFs. The frequency response of the QTFs as a function of the driving frequency of the function generator were recorded by a personal computer (PC). The Lorentzian function was used to fit the frequency response curve and obtain the QTF resonance frequency *f_0_*, the full-width at half-maximum (FWHM) *Δf* of the signal response and equivalent resistance *R*. In this way, the Q factor was calculated as the ratio between the resonance frequency *f_0_* and *Δf*. In Table 2, the resonance frequencies and related Q-factors and electrical resistances measured at atmospheric pressure are listed, together with the corresponding theoretical resonant frequencies *f_th_* estimated with Equation (2) using values *E* = 0.72·10^11^N/m^2^ and *ρ* = 2650 kg/m^3^.

The results show that the Euler–Bernoulli beam theory can predict the resonance frequency of the fundamental flexural mode of the QTF with a good accuracy. For prong sizes (*w* and *L*), the uncertainty was less than 1%, leading to an uncertainty of the predicted resonance frequency less than 1 Hz. The precision in measured resonance frequency was in the mHz range. The discrepancies between experimental and theoretical values were mainly due to the damping of the gas and the additional weight due to the electrode layers [22]. The electrical resistance was calculated as the ratio between the QTF piezocurrent and the excitation voltage at the resonance, since, in this condition, the QTF performs as a pure resistor. The obtained results show that QTF#2 exhibits the highest Q-factor and the lowest electrical resistance. However, even if the resonance frequency of QTF#3 was 4.5 times lower than QTF#2, the quality factor was only 1.8 times lower. 

## 3. Quartz-Enhanced Photoacoustic Sensor

**Figure 3:**
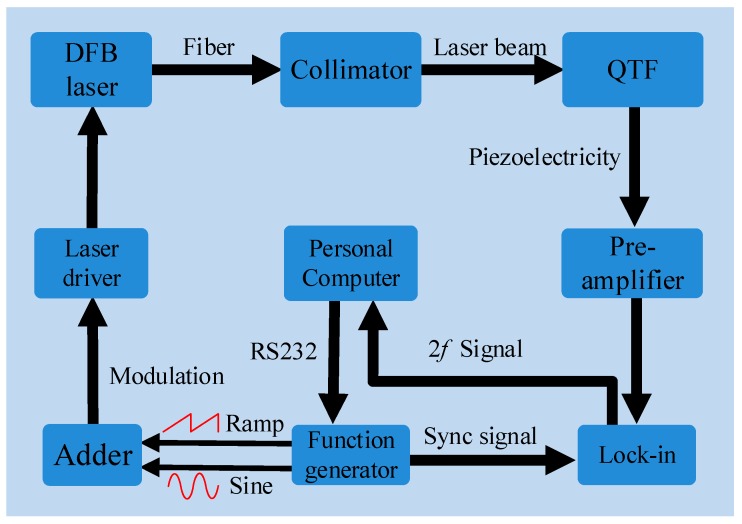
Schematic diagram of the quartz-enhanced photoacoustic spectroscopy (QEPAS) experimental setup. The double channel function generator produces ramp and sine signals to tune and modulate the laser wavelength, respectively. QTF: quartz tuning fork, DFB lasers: distributed feedback lasers, Lock-in: lock in amplifier.

In this section, a QEPAS sensor system for water vapor detection was described. The laser beam focus position between two QTF prongs as well as the laser modulation depth were investigated in detail to optimize the sensor performance. The schematic diagram of the experimental setup is depicted in Figure 3. 

A 1.37 µm near-infrared fiber-coupled distributed-feedback (DFB) diode laser was employed as the excitation source to detect water vapor (H_2_O) and to generate the QEPAS signal. The wavelength of the diode laser can be coarsely and finely tuned by changing the temperature and the injected current, respectively. A 2 channel arbitrary waveform function generator (Tektronix AFG3102) was used to produce a ramp signal with a frequency of 10 mHz to tune the emission wavelength of the diode laser. A sine signal with the frequency of *f_0_/2*, where *f_0_* corresponded to the resonance frequency of the QTF, was added to the laser driver. The output laser beam from the pigtail was focused between the prongs of the QTF by means of a fiber-coupled focuser. An aluminum enclosure, equipped with two windows, was realized in order to accommodate as well as conveniently interchange the custom QTFs. The sound wave induced by the photoacoustic effect occurring in the absorbing gas drives the vibration of the QTF prongs. The electric signal generated by the piezoelectric effect in quartz was detected using a transimpedance amplifier with a feedback resistor of 10 MΩ and a lock-in amplifier, which demodulated the signal at the QTF resonance frequency. The concentration of H_2_O in the lab air was determined by direct absorption spectroscopy. The experiment was conducted at atmospheric pressure of ~700 Torr (9.3 × 10^4^ Pa) and a room temperature of ~25 °C. 

### 3.1. Optimization of Laser Beam Position

**Figure 4:**
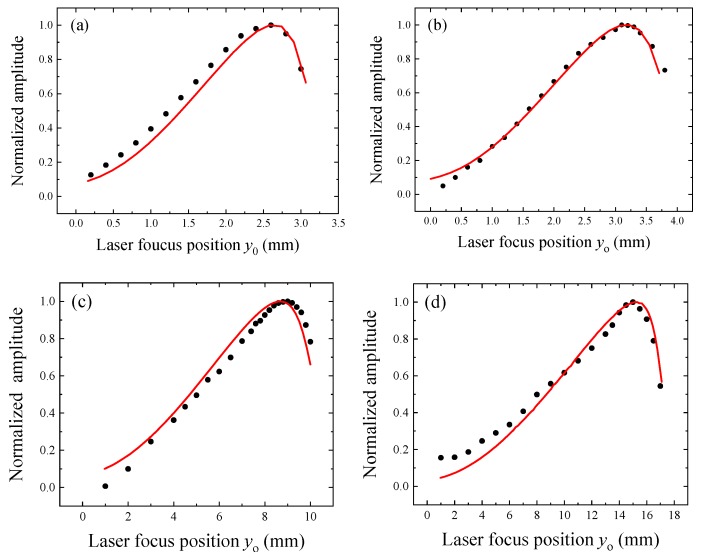
(**a**–**d**) Normalized QEPAS signal amplitudes as the function of laser focus position *y*_0_ measured for QTFs #1 (panel a), #2 (panel b), #3 (panel c), and #4 (panel d). Black dots represent the experimental data and the red lines represent the theoretical curve calculated by the numerical method.

The *y*_0_ denotes the distance between the laser focus position and the junction between the prong clamped end and the quartz support, as shown in Figure 1a. A simplified theoretical model which considers the total momentum of a pressure force acting on the two prongs of the QTF have been reported in References [7,8], which assumes that the sound wave located between two prongs is a point-source and the intensity of the pressure wave decreases as the inverse of the distance. The dependence of the QEPAS signal strength (which is proportional to the total momentum generated by the pressure wave) as a function of *y*_0_ was experimentally investigated. The position of the optical fiber focuser was adjusted by an *xyz* linear translation stage with a resolution of 0.01 mm. The waist diameter of the focused laser beam was ~100 µm. The QEPAS signal amplitudes normalized to the related highest values are plotted in Figure 4 as the function of *y*_0_. 

The optimum laser focus positions maximizing the QEPAS signal were extracted and are listed in Table 3, together with the predicted values estimated using the theoretical model reported in Reference [7]. The numerical method and experimental results were well consistent, indicating that the mathematical model was able to predict the optimum laser focus position.

The slight discrepancy between the theoretical and experimental results can be attributed to the following reasons: (i) the acoustic pressure wave generated by the interaction of the laser beam with the trace gas was modeled as a wave that propagates in free-space, thus its wavefront was assumed to not be distorted by the interaction with the QTF; (ii) the QTF was modeled as a system of two non-coupled cantilevers, vibrating independently from each other; and (iii) the energy damping of the vibration prong and the additional weight due to the electrode layers were both not included in the model.

### 3.2. Optimization of Laser Modulation Depth

**Figure 5:**
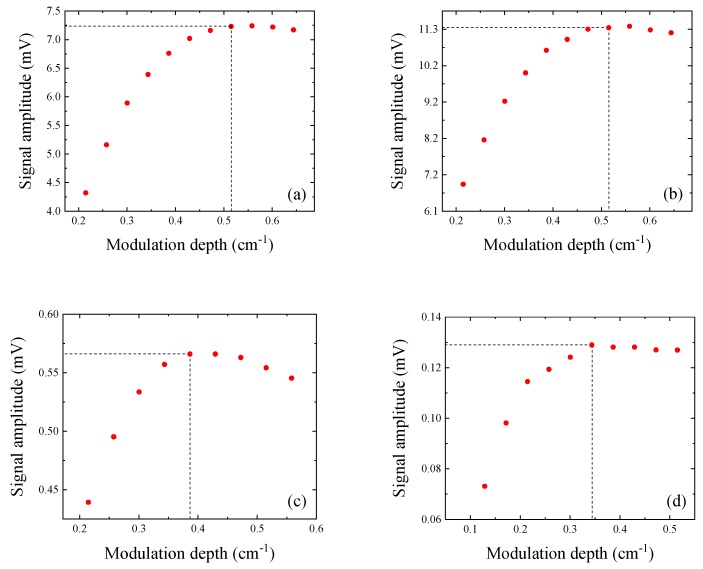
2*f* QEPAS signal amplitude as a function of modulation depth for QTF#1 (**a**), QTF#2 (**b**), QTF#3 (**c**), and QTF#4 (**d**).

When the wavelength modulation approach is used, the modulation depth describes how much the laser wavelength varies around its mean value. According to the theory of wavelength modulation spectroscopy, the laser modulation depth maximizing the QEPAS signal should be optimized for each laser current modulation frequency [23]. Hence, the laser wavelength modulation depth was varied from 0.2 cm^−1^ to 0.6 cm^−1^ for each employed QTF, by varying the current modulation amplitude. The 2f-QEPAS peak signal as a function of the modulation depth is reported in Figure 5, for all investigated QTFs. 

With QTF#1 and #2 having the same resonance frequency of ~32.7 kHz, the optimal modulation depth was ~0.52 cm^−1^ for both QTFs. For QTF #3 (*f_0_* = 7.21 kHz) and #4 (*f_0_* = 2.86 kHz), the optimal modulation depths were 0.39 cm^−1^ and 0.35 cm^−1^, respectively. These results demonstrate that the modulation depth maximizing the 2f-QEPAS signal increases as the laser wavelength modulation increases. This affirms that in QEPAS, the modulation depth is a parameter related not only to the gas absorption line to be detected, but also to the resonance frequency of the QTF employed.

## 4. QEPAS Sensor Performance

**Figure 6:**
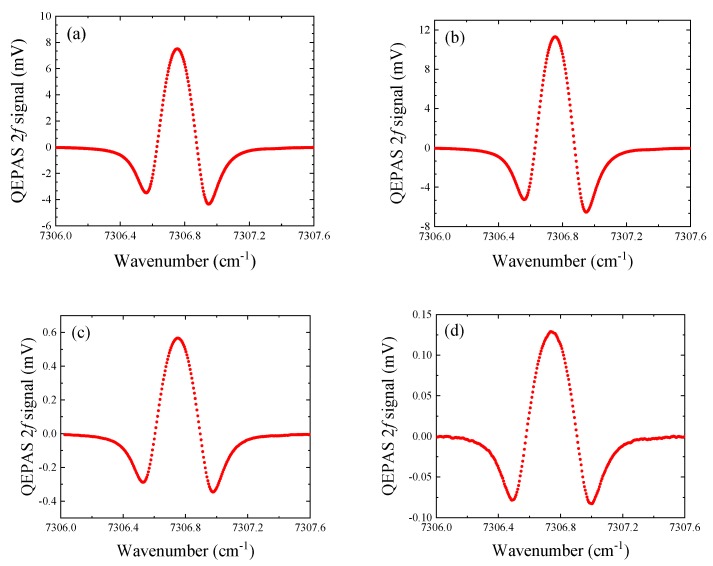
Optimized 2*f* QEPAS signals. (**a**–**d**) QTF#1, #2, #3, and #4, respectively. The laser wavelength was targeted at the 7306.75 cm^−1^ H_2_O absorption line.

The water vapor in ambient air was detected using the QEPAS sensor depicted in Figure 3 and by swapping the four QTFs. The laser injection current was tuned from 80 mA to 120 mA, corresponding to wavenumber spanning from 7306 cm^−1^ to 7307.6 cm^−1^, in order to cover the 7306.75 cm^−1^ H_2_O absorption line having a line strength of 1.8 × 10^−20^ cm/molecule [24]. The obtained QEPAS 2*f* scans are shown in Figure 6. For each QTF, the conditions for optimum laser focus position and optimum modulation depth have been employed. The time constant and filter slope of the lock-in amplifier were set to 1 s and 12 dB/Oct, respectively. 

The peak values of spectral scans for QTF#1, #2, #3, and #4 were 7.5 mV, 11.3 mV, 0.57 mV, and 0.13 mV, respectively. The 1σ noise was calculated from the standard deviation of the QEPAS signal when the laser emission wavelength was tuned away from the H_2_O absorption line. In this way, the minimum absorption coefficient *α_min_* can be estimated together with the normalized noise equivalent absorption (NNEA), calculated by normalizing *α_min_* to the laser power ($P_{L}$) and the detection bandwidth ($\Delta f_{L}$) according the relation:

(3) $$NNEA=\frac{\alpha_{min}P_{L}}{\sqrt{\Delta f_{L}}}$$

All these parameters are summarized and listed in Table 4.

By comparing QTF#1 and QTF#2, the 2*f* signal peak obtained by QTF#2 was 1.5 times larger than QTF#1. Since the Q factor of QTF#2 was 1.6 times larger than that of QTF#1 and they shared the same resonance frequency, this affirms that the QEPAS signal was strongly dependent on the QTF quality factor, as predicted by Equation (1). The 2*f* signal peaks obtained by the two custom QTFs were one order of magnitude lower than that of commercial QTFs. In particular, the 2*f* signal peak obtained with QTF#2 was ~80 times higher than that obtained by QTF#4. This huge discrepancy in terms of performance can be explained based on the following arguments. First, the Q-factor of commercial QTFs were higher than those of custom QTFs. The Q-factor depends on all the energy dissipation mechanisms occurring in a vibrating prong of a QTF. The main contributions are due to the damping by the surrounding fluid, the interaction of the prong with its support and thermo-elastic damping. All these loss mechanisms strongly depend on the QTF prongs’ size [25,26]. Hence, the quality factor will depend on the prong geometry, as well as the fundamental resonance frequency. This suggests that the quality factor and the resonance frequency must be related to each other, at different prong geometries. Second, the conversion efficiency *ε*(*P, f*_0_) can be negatively affected when the resonance frequency goes down to a few kHz. Third, the electrical resistance of custom QTFs are much higher than commercial QTFs, suggesting that the QTF response at the resonance frequency is lower as well as the piezoelectric charge collection efficiency. To confirm these two last points, the conversion efficiency *ε*(*P,f*) should be investigated as a function of the modulation frequency, by using different QTFs sharing almost the same Q-factor and electrical resistance. Since the focused beam size (~100 µm) was lower than the smallest prongs spacing (200 µm for QTF#1), the photothermal noise due to the portion of light hitting the QTF can be neglected for all investigated QTFs. Hence, the 1σ noise level was dominated by the thermal noise that can be expressed by [27]:

(4) $$1\sigma \;noise=\sqrt{\Delta f_{L}}R_{f}\sqrt{\frac{4KT_{e}}{R_{f}}}$$

where *K* = 1.38·10^−23^ J·K^−1^ is the Boltzmann constant, *R_f_* = 10 MΩ is the feedback resistor of the transimpedance amplifier, and *T_e_* = 300 K is the QTF temperature. Hence, for custom QTF#3 and QTF#4 having significantly higher electrical resistance with respect to commercial QTFs, the 1σ noise level is reduced.

## 5. Conclusions

In this work, the performance of four QTFs were compared when employed as acousto-electric transducers in a QEPAS sensor. Standard QTF#1 and #2 had the same resonance frequency of 32.7 kHz, while the Q factor value of QTF#2 was about 60% higher than QTF#1. The custom QTF#3 and QTF#4 had lower resonance frequency (7.2 KHz for QTF#3 and 2.9 KHz for QTF#4) and larger prong spacings (0.8 mm for QTF#3 and 0.7 mm for QTF#4) with respect to commercial QTFs. The quartz wafer used to etch custom QTFs was 0.25 mm, thinner than that of standard QTFs (0.33 mm). The fundamental resonance frequencies can be easily predicted using the Euler–Bernoulli beam theory. Regarding the acousto-electrical properties, the quality factor of custom QTFs were lower and electrical resistance significantly higher with respect to standard QTFs. Performance of the QEPAS sensors based on these four QTFs were evaluated using a near-infrared DFB diode laser to detect the H_2_O in ambient air. The laser focus position along the QTF axis was optimized. The optimal laser modulation depth was found to be dependent on the modulation frequency. Despite custom QTFs allowing a reduction of the modulation frequency, the best results were obtained using standard QTFs. This demonstrates that for any QTF resonance frequency, the quality factor must be kept as high as possible and the electrical resistance as low as possible in order to guarantee high QEPAS performance. Finally, the ultimate noise level was dominated by the thermal noise which was determined by the electrical resistance of the QTF and the detection bandwidth. The performance of a QEPAS system may be improved by employing a digital lock-in amplifier based on a field programmable gate array [28].

## Figures and Tables

**Table 1:** The geometric parameters of four different QTFs. *W, L,* and *T* are the prong width, length, and thickness, respectively, as defined in Figure 1a. *g* is the spacing between two prongs.

QTF	Geometric Parameters			
	*W* (mm)	*G* (mm)	*L* (mm)	*T* (mm)
#1	0.38	0.20	3.00	0.33
#2	0.58	0.29	3.73	0.32
#3	0.90	0.80	10.0	0.25
#4	1.00	0.70	17.0	0.25

**Table 2:** The resonance properties measured for four different QTFs. *f_0_*: resonance frequency, *Q*: Q-factor value, *R*: equivalent resistance. *f_th_* is the predicted resonance frequency using Equation (2).

QTF	Electric Parameters			
	*f_th_* (kHz)	*f_0_* (kHz)	*Q*	*R* (kΩ)
#1	32.55	32.75	8900	208
#2	32.10	32.77	14,300	93
#3	7.58	7.21	6900	351
#4	2.91	2.86	5800	721

**Table 3:** Comparison of theoretical estimation of optimum laser position given by the numerical model reported in Reference [7] and results obtained experimentally.

Method	QTF#1	QTF#2	QTF#3	QTF#4
Theoretical (mm)	2.6	3.1	9.0	15.0
Experimental (mm)	2.6	3.2	8.7	15.2

**Table 4:** 1σ noise level, minimum absorption coefficient (*α_min_*), and normalized noise equivalent absorption coefficient (NNEA) of the QEPAS sensor, for each QTF employed in this work.

QTF	Peak Signal (mV)	1σ Noise (µV)	α_min_ (cm^−1^)	NNEA (W·cm^−1^·Hz^−1/2^)
#1	7.5	3.1	1.2 × 10^−5^	4.5 × 10^−7^
#2	11.3	3.9	1.0 × 10^−5^	3.8 × 10^−7^
#3	0.57	2.4	1.2 × 10^−4^	4.5 × 10^−6^
#4	0.13	1.6	3.6 × 10^−4^	1.4 × 10^−5^
